# Comorbidity of Crimean-Congo Hemorrhagic Fever and COVID-19

**DOI:** 10.1590/0037-8682-0429-2021

**Published:** 2021-09-24

**Authors:** Ayse Albayrak, Handan Alay, Sibel Iba Yilmaz

**Affiliations:** 1 Ataturk University, Faculty of Medicine, Department of Infectious Diseases and Clinical Microbiology, Erzurum, Turkey.; 2 Republic of Turkey Ministry of Health, Erzurum City Hospital, Department of Infectious Diseases and Clinical Microbiology, Erzurum, Turkey.

**FIGURE 1:**
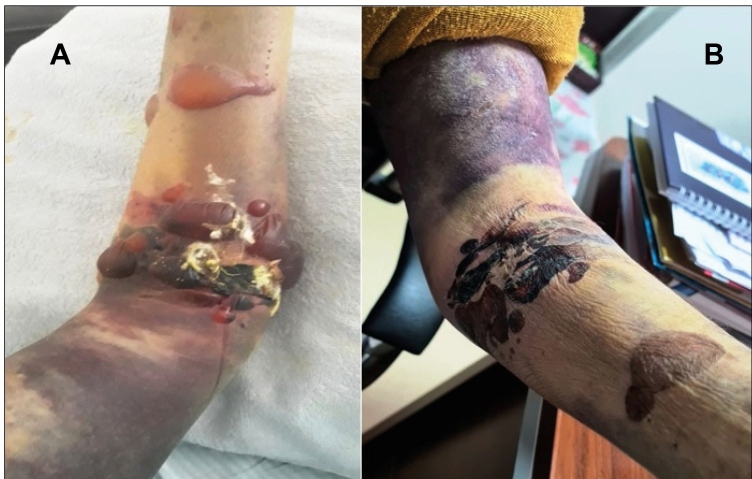
**(A)** Subcutaneous hematoma, diffuse ecchymosis, and bullous lesions on the patient’s left arm; (**B)** regression of the lesions after treatment.

A 61-year-old woman presented to the emergency department with fever, weakness, headache, and cough that had persisted for one week. Her pulmonary saturation was 87%, and her hemogram, biochemistry, and bleeding parameters were normal. The pulmonary tomography report described ‘several areas of linear atelectasis in the basal parts of both lungs’. The patient’s SARS-CoV-2 polymerase chain reaction (PCR) test was positive, and she was started on low molecular weight heparin (LMWH) 0.6 mL/day and dexamethasone 6 mg/day therapy. On the fifth day of follow-up, her WBC was 2.97X10^3^/µL (lymphocyte count, 510); platelets, 45X10^3^/µL; aspartate-aminotransferase, 537 U/L; alanine aminotransferase, 341 U/L; lactate dehydrogenase, 590 U/L; ferritin, 1650 ng/mL; and D-dimer, 35,200 ng/mL. No shortness of breath or other respiratory symptoms were observed. However, she described having removed a tick from her body approximately a week before. Hematoma, diffuse ecchymosis, and bullous lesions developed on the left arm to which the LMWH had been administered (Figure 1A). The patient’s Crimean-Congo hemorrhagic fever (CCHF) PCR test results were positive. LMWH therapy was stopped, and supportive therapy was initiated for CCHF. The hematoma on the arm resolved during follow-up (Figure 1B).

CCHF is a zoonotic viral disease endemic in Turkey. While diagnosis is relatively easy in patients with a tick bite history, it can be difficult in patients without one^1^. While CCHF progresses with hemorrhage, COVID-19 causes thrombosis^2^. Similar symptoms and laboratory findings can be seen in both diseases. CCHF must be considered in regions where it is endemic during the COVID-19 pandemic. 
